# Tenure mix: apart or together? Home-making practices and belonging in a Dutch street

**DOI:** 10.1007/s10901-015-9493-y

**Published:** 2015-11-07

**Authors:** Peer Smets, Karin Sneep

**Affiliations:** Department of Sociology, Faculty of Social Sciences, VU University, De Boelelaan 1081, 1081 HV Amsterdam, The Netherlands

**Keywords:** Home, Home-making, Belonging, Tenants, Owner-occupiers, Micro-setting

## Abstract

This paper discusses home-making practices and senses of belonging in a street in a disadvantaged neighbourhood in the south of the Netherlands. The local tenure mix of tenants and owner-occupiers offers insight into the role class and ethnicity play in social mixing. Therefore, attention is paid to narratives and the informal organisation of different living spaces and territory-making practices. Here, the domestic space could be experienced as a vehicle of intimacy and sociability, or conversely as encouraging alienation. Such practices, in combination with length of stay result in mechanisms of inclusion and exclusion. The insights derived from this study will contribute to the theoretical discussion on home-making practices and belonging.

## Introduction

In this period of globalisation, daily practices of local home making and feeling at home are considered important. This is a phenomenon relevant not only for migrants but also for local residents who settle in a new place where it is not always clear what is private, public or communal. Many neighbourhood residents try to create a sense of home; however, home is a concept with different meanings (for overviews see, e.g. Antonsich 2010; Mallet 2004; Rykwert 1991), with only those that are relevant to this study given below. It can have a physical component such as shelter with walls and a roof, but also social, cultural, psychological and emotional components (e.g. Mallet 2004; Duyvendak 2011; Moore 2000; Rykwert 1991). It is important to realise that it is human beings who attach significance to the concept of the home, which can be place and time dependent (Easthope 2004). Home can also be seen as a private place where one can realise desires and expectations of life, but can also relax and enjoy free time. Apart from a kind of comfort, home is a space where independence, freedom and safety can be expected. People feel at home once ‘they have control, or at least exercise a degree of control, over a space’. Such control over a space implies also ‘the ability to take or exercise some control over one’s life’ (Parsell 2010, p. 3).

Although there are multiple interpretations of the concept of home, Moore (2000, p. 208) stresses that ‘the concept of *home* has to be examined in terms of its parts as well as a whole, mindful that to focus strongly on one part, it is possible to lose sight of the whole concept itself.’ In addition, Moore (2000, p. 213) argues that home is mainly studied from the perspective to its physical form, while at the same time this feature of home is relatively unexplored. Another important aspect is the context in which the house is placed; areas with similar housing and people, or mixed populations. Once it concerns mixed populations, residents, especially those from the middle classes of society, tend to draw specific boundaries between them and others (Atkinson 2006; Watt 2009).

Home making can be different for different income groups living in the same neighbourhood. Once these classes live together in a neighbourhood, they often have other tenures; rental and owner-occupiers housing (see, e.g. Baker 2013; Boersma et al. 2013; Smets and Hellinga 2014). Once people reside somewhere for a longer period, they can get more attached to that particular place and consequently feel more responsible for their living environment. Such a responsibility can be found among owner-occupiers who possess the premises and have the means available for maintenance and improvement of their house (Riger and Lavrakas 1981, p. 56). Another incentive for owner-occupiers to invest in their houses is the expected increases in their value (e.g. Herbert and Belsky 2008; Shlay 2006, p. 513). Meanwhile, a further assumption is that tenants tend to invest less in their house and take less care of their living environment, with regards to garden, street and neighbourhood. Moreover, rubbish and noise irritation appears to be more common among tenants in comparison with owner-occupiers (Ket and Papa 2001, p. 5). One should realise that higher-income groups do not necessarily reside in owner-occupier housing. They can still reside in social housing while earning a high income in spite of being able to pay more for housing. Moreover, those residing in rental housing will not always refrain from taking care of their home even if their means are limited.

Blue print notions of the behaviour of residents with different types of tenure impact upon mixing policies to a large extent. A mix of tenants and owner-occupiers often implies a mix of income groups. As income is related to ethnicity, attention should also be paid to inter-ethnic contact between groups and individuals. Social mixing in deprived neighbourhoods does not imply that middle- and lower-class residents have contact; they are more likely to have no contact, creating a socially ‘tectonic’ situation (Robson and Butler 2001; Butler 2007, p. 173). Social mixing research mainly focuses on middle-class residents. In this respect, Watt (2008) remarks that the working class fundamentally plays a backstage role.

In policy circles on social mixing, it is assumed that physical proximity of different classes would create social capital, which in turn would create inclusion and access to resources. Scientific literature questions such optimistic impacts of social mixing of different income groups in neighbourhoods (e.g. Lees 2008; Musterd and Andersson 2005; Smets and Den Uyl 2008; Smets and Hellinga 2014). If social mixing goes together with a tenure mix, it can be seen as a way of reconquering the city in favour of owner-occupiers (Smith 1996; Uitermark and Duyvendak 2008), colonising the city (Atkinson 2006), or reducing spatial inequalities in access to facilities and services (Smets and Salman 2008). Such changes go together with improving urban liveability to enable competition in a globalised knowledge-based economy (Florida 2003).

For positive mutual contact and communication, one requires an attitude of openness, curiosity and self-awareness. Positive experiences by contacting the other can also lead to the development of affective bonds, but misunderstandings and incomprehension caused by ethnic difference can lead to the avoidance or severance of contact, even when it concerns greeting only (Tennekens 1994). However, living close to others with different ethnic background does not imply that one can easily overcome ethnic and class divisions in social networks (e.g. Atkinson 2006; Blokland 2003; Smets and den Uyl 2008). Even when middle-class residents judge a diverse population positively, they can separate themselves from other residents by sending their children to separate schools (Butler 2003; Galloway et al. 2014; Smets and Salman 2008).

To obtain insight into the dynamics of social mixing, we investigated an urban setting in the south of the Netherlands. Here, social tenants and owner-occupiers reside in close proximity, and the initial strategy was for interaction or mingling between the two. It is important to also take into account how different groups of residents create a home and a sense of belonging. The research question is: ‘What role does home making play in the everyday practices of social mixing of tenants and owner-occupiers in Diversity Street, in a medium-sized city in the south of the Netherlands?’ This paper is built up as follows: firstly we offer a discussion involving theoretical insights into home, home making and senses of belonging, closely followed by a methodology section and a description of the research setting. We regard the street as a natural laboratory; we divide it into five different blocks with each section containing a different pattern of interaction. The behaviour and opinions of tenants and owner-occupiers both within and between clusters will be discussed. Finally, this paper will be wrapped up in a conclusion.

## Home and belonging

For many people, home is a place of belonging, intimacy, security, relationship and selfhood. Through their investments in their home people develop their sense of self and their identity. Others experience alienation, rejection, hostility, danger and fear “at home”. Houses are the material structures that provide the scaffolding for emotional investments, social relations and meanings of everyday life (Dowling and Mee 2007, p. 161).

Home-making practices—which can also be applied to sections of neighbourhoods as will be done in this paper—take place against the backdrop of permanency and movement, staying and leaving, continuity (practices of everyday life) and discontinuity (changes which threaten everyday practices) (Mallet 2004, p. 79; Martucci 2013; Reinders and van der Land 2008, p. 7).

The result of home and home making can contribute to a sense of belonging; a concept of analysis increasingly used in social sciences (e.g. Savage et al. 2005; Watt 2009; Duyvendak 2011; Smets and Watt 2013; Watt and Smets 2014).

To look into senses of belonging, Antonsich (2010) proposes the use of a combination of place belongingness and the politics of belonging. Place belongingness refers to belonging as a personal intimate feeling of being at home in a specific place. Everyday living can be linked with the possibility and impossibility of movements within and outside the neighbourhood. In this respect, Moore (2000, p. 213) emphasises that we ‘need to focus on the ways in which home disappointments, aggravates, neglects, confines and contradicts as much as it inspires and comforts us’ (emphasis by Moore).

The general concept of place belongingness is the focus of many studies, but the focus in this research will be on place belongingness at the neighbourhood level. Savage et al. (2005, p. 12) consider belonging ‘a socially constructed embedded process in which people reflexively judge the suitability of a site as appropriate given their social trajectory and their position in other fields.’ The place where people reside plays an important role for finding a localised notion of being at home in an increasingly globalised world; however, place attachment and social inclusion connected to place are no longer self-evident (ibid., pp. 12–29).

Watt (2009, p. 4) stresses the need ‘to explore the moral judgements vis-à-vis class as well as racialised “others” that underpin residential space, place images, and attachment’. Underlying these moral judgements are relational notions of taste and distaste (Bourdieu 1979, p. 56), which are reflected in narratives of belonging. Watt (2009) also shows how processes of class distinction can be used for studying the middle classes’ employment of exclusion mechanisms in London’s eastern suburban fringe. Here he found that some residents had a spatially selective narrative of belonging, which not only refers to a specific space where people reside, but also includes part of a wider locality. These people enjoy living in a specific area, which provides opportunities for group-forming with other middle-class members of society. The positive image of such a space is, however, not shared by the wider area.

Watt’s notion of selective belonging can be linked with the micro-setting of Kusenbach (2008, p. 323); ‘small niches of community that sometimes flourish within subsections of urban street blocks’. This idea can relate to the combination of a shared-built environment—for instance, walls, adjacent properties, common access to the premises through, for example, a security gate, shared facilities and courtyards—and where practical use of the environment provides insight into visible private and semi-private routines. This kind of micro-setting may be characterised by different lifestyles and/or diversities with regard to ethnicity, religion and age. In such a setting, trust and mutual dependency play an important role. Moreover, the interactions and relationships between neighbours are characterised by passive contact, sociability, proactive neighbouring and friendships where collective rituals and representations are reflected in informal gatherings, nicknames and the reputation of places (pp. 232–235). The focus on a micro-setting makes it possible to look into:

terms of day-to-day living. This is particularly likely when there are marked economic or ‘lifestyle’ disparities between residents, which can generate discomforting experiences of neighbouring (Rose 2004, p. 281).

Insight into day-to-day living, social mixing and the mutual image-building of neighbourhood residents all demonstrate and impact upon their sense of place belongingness. When newcomers settle in a neighbourhood, they are often strangers to the already established residents. Such strangers can be physically nearby but socially distant, a reflection of differing lifestyles, habits and values and manifest in us-and-them configurations between the physically nearby but socially distant groups (e.g. Elias and Scotson 2001; Savage et al. 2005; Smets and Kreuk 2008; Galloway et al. 2014).

The second type of belonging as proposed by Antonsich (based on Yuval-Davis 2006) is the politics of belonging as a ‘discursive resource which constructs, claims, justifies, or resists forms of socio-spatial inclusion and exclusion’, which goes hand in hand with the construction of symbolic and physical boundaries regarding the local community. Here, belonging is an individual or collective process of rejection, violation, transgression or negotiation, whereby belonging can be claimed or granted. Those who claim belonging also claim the right to stay (p. 14). However, to belong, a person often has to adjust or assimilate to the language, values, behaviour, culture and religion of the dominant group (Yuval-Davis 2006, p. 209). Attention will now be paid to the methodology used for this research.

## Methodology

The theoretical concepts derived from the literature studied should be seen as sensitising concepts which enable the researcher to look into the research setting with an open mind (Blumer 1954). Consequently, we started with as few pre-conceived ideas as possible, but gradually refined general concepts to fit the empirical situation under study. Through this procedure, theoretical insights are allowed to emerge gradually from the data in dialogue with the collection of new information (Glaser and Strauss 1973).

To gain insight into the social relationships and the sense of belonging attributed to owner-occupiers and social housing tenants, a series of ‘participant’ observations on the built environment and people’s behaviour, informal chats and in-depth semi-structured interviews were conducted with 11 tenants and 10 owner-occupiers, in the period from April to June 2008. During the interviews, questions were asked about the personal experiences and knowledge of other residents. Interviewees were selected from the whole street under review and were approached by ringing their doorbells. It soon became evident that many tenants of migrant^1^ origin were not able and/or unwilling to be interviewed and as the owner-occupiers are mainly ‘white’, this led to a ‘white’ bias. This bias may be strengthened further by the fact that the authors of this article are of ‘white’ native-born origin. Such a bias was minimised by incorporating observations, allowing us to compare our findings with the research material derived from the interviews and therefore providing more in-depth insights. One should also be aware that despite the small sample, a rich array of material was gathered. The first interviews provided insight into the interaction between people. Subsequent interviews and informal talks largely reaffirmed the findings obtained from previous interviews. Moreover, observations contributed substantially to understanding everyday practices of many residents.

All data obtained were processed by means of inductive content analysis in which the insights from one interview were applied to the following. In other words, a process of cumulative knowledge building took place. All data were coded, analysed and interpreted using the constant comparative method (Glaser and Strauss 1973, pp. 101–115). However, the number and types of groups derived from the collected data could only be evaluated once all the data had been gathered. From here, we were able to develop each major category (Glaser and Strauss 1973: 50) in different micro-settings. Consequently, not all micro-settings offer similar representatives. It appears that the offered up perspectives referring to other residents mainly pertain to those living nearby. Although the research findings are not representative, this exploratory study provides insights into dissimilar patterns of belonging in different micro-settings.

Finally, one should realise that the names of the interviewees and streets are fictitious in order to protect the privacy of these residents. The names of the clusters are made up by the authors, except the White and the Migrant Block, which have emic names. The other block names are derived from our research material, which offer insight into the differing lifestyles of the researched population and are inherently linked with the concept ‘belonging’. Below greater attention will be paid to Diversity Street, the geographical area in which the research took place.

## Diversity Street

Diversity Street is located in a medium-sized city in the south of the Netherlands, which consists of former small settlements that grew together as a direct result of an expanding wool and textile industry that flourished until the second part of last century.

After the Second World War, the urban population increased enormously but was confronted with the closure of many textile factories. This resulted in the development of many new neighbourhoods that are currently considered to be disadvantaged. Here, we find low-income groups including non-Western migrants and long-term unemployed. Diversity Street is located in one of these disadvantaged post-war neighbourhoods.

Diversity Street has different types of mixed rental and owner-occupied housing, which makes it an interesting setting for this study. In the 1990s, the original flats on the edges of the street were demolished and replaced by private owner-occupied housing. Moreover, some tenants were offered the chance to buy their rental property, consequently leading to a mixture of both owner-occupied and rental housing.

**Fig. 1:**
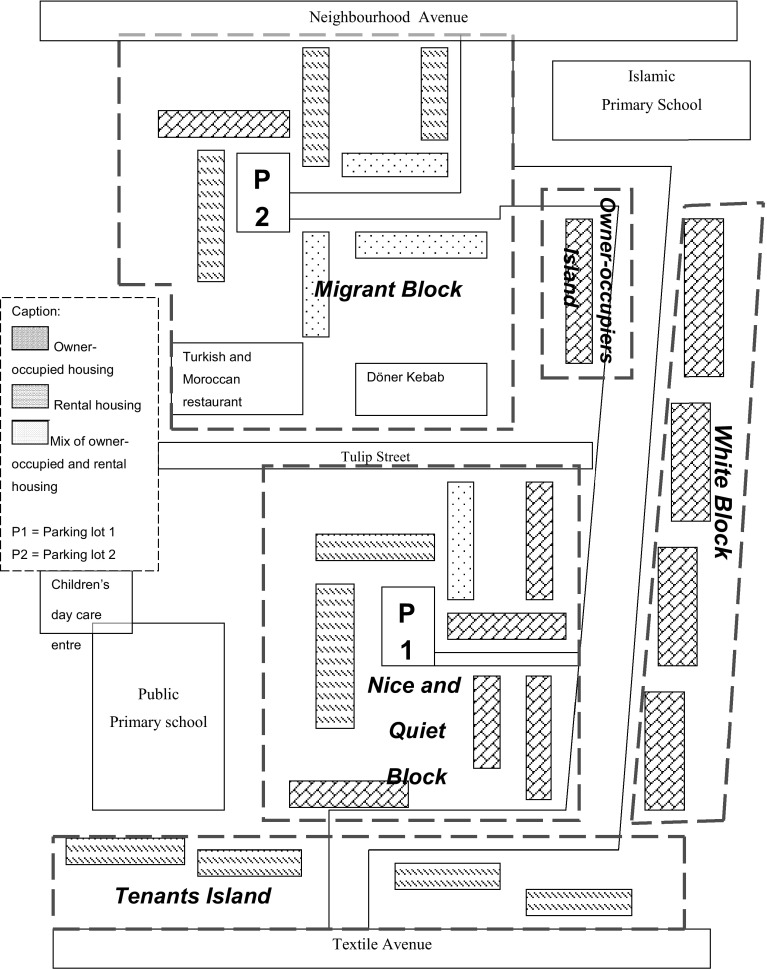
Diversity Street

One should realise that the blocks chosen as clusters in this research (see Fig. 1) were determined on the basis of physical parameters, such as the type of housing and demarcation of streets. The clusters—characterised by different constellations of tenants and owner-occupiers—are: (1) the White Block, (2) the Nice and Quiet Block, (3) Tenants Island, (4) Owner-occupier Island and (5) the Migrant Block.

### White Block

Houses in the White Block—the most recently constructed houses in the street—have white stone walls and well-maintained gardens. In 1992 these houses were constructed on the location of a former apartment complex. Many contemporary owner-occupiers settled here such as just-married young urban professionals and yuppies. Once these new owner-occupiers had children, they became young urban professional parents or ‘yupps’, a term borrowed from Karsten (2003). From 1992 onwards, a community feeling developed among the residents. Stephan (39)—one of the residents—reports:

We have contacts in this street. I mean, a few people came to live here 16 years ago. (…) We also returned here then, just married (…). Contact was frequent back then and natural. All social interaction was pleasant. Mostly that contact still exists. Some people have moved (…). They wanted something else, but in general the contact is good. When new people move in, I usually give them flowers to welcome them to the neighbourhood. Anyone can come here and will be accepted, but there must be a mutual spin off.

The owner-occupiers belong to the ‘white’ middle class (aged 35–55) who have mutually weak or strong ties, which can be reflected in their relationships with one another, such as being acquaintances or friends. The men have full-time jobs, and the women work mostly 2–3 days a week enabling them to combine childcare and work.

These houses were bought in the 1990s when house prices were relatively low. During the period of research, residents reported being easily able to save money for a second car or vacations abroad. Others invested in their homes by upgrading them. Comfort of living and day-to-day conveniences with investments in personal living space or home improvements was considered as an investment for the future.

The desire of owner-occupiers for having a good reputation in the neighbourhood is manifest in a shared lifestyle where neatness and attention is paid to the appearance of their homes and gardens. Moreover, they are members of a residents association linked to the White Block, which organises activities for its members such as a treasure hunt, a cycle tour, an annual street barbecue along with festivities linked to national events. The association also maintains a website publishing notifications of its activities and festivities in the neighbourhood. The ‘whites’ also regularly help each other out by exchanging information on, for example, finding a new window cleaner or a burglary that took place nearby.

Although the community feeling in the White Block is well developed, the owner-occupiers spend most of their time outside the neighbourhood. These same residents have put their children into schools outside the neighbourhood because the local ‘black’ primary schools are considered inferior. Moreover, children of the White Block rarely play with children from the other clusters. The parents of the ‘white’ children are engaged in free-time activities and contacts within their own block and outside the neighbourhood.

When the residents of the White Block bought their house, many considered the location of their house as ‘good’ and the neighbourhood ‘quiet and pleasant to live in’. Moreover, most saw the two primary schools and the children’s day care centre as suitable for their future children. However, once they had children, these schools were no longer considered good enough. Furthermore, the neighbourhood was increasingly seen as unattractive due to its large number of immigrants.

To sum up, here one sees that place belongingness plays an important role: home making and living in a ‘pleasant’ cluster with people of a similar lifestyle and ethnic background whilst at the same time disliking the broader neighbourhood and its schools. Once these highly educated residents became concerned with passing on their cultural capital to the next generation, the interviewees developed an unwillingness to do so in a ‘mixed’ neighbourhood. For these residents, their block is a home characterised by the continuity of daily practices of life; maintenance of the physical and social environment in their cluster goes together with patterns of mobility with regards work, school, shopping and free-time activities outside the neighbourhood. Place belongingness is only experienced within their own cluster, where the local politics of belonging are strongly developed among the residents. Although most residents set clear physical and social boundaries of inclusion for insiders and exclusion for outsiders, they think that the neighbourhood is a good reflection of the world today; yet they refrain from mingling.

### Nice and Quiet Block

This cluster shows how owner-occupiers and tenants in mainly single-family houses can and do live together as ‘good’ neighbours. Although many tenants moved out, other residents—tenants as well as owner-occupiers—have lived in this block since its construction in 1972 and are happy to remain. The owner-occupiers are mainly ‘white’ and the tenants include many non-Western migrants. However, in the mixed blocks where owner-occupiers and tenants reside simultaneously, both groups of residents are predominantly ‘white’. Residents of the tenant blocks are of mixed ethnic origin and have lived there for a long time. Most of the houses are not directly connected to the main street but have their entrance adjacent to a pavement or parking lot.

Children have grown up and moved out, visiting occasionally, with their own children, their parents. Residents have differing opinions in relation to the buying or renting of a house. Tenants stress that the pleasure of life is uncomplicated by expensive time-consuming repairs and maintenance when renting, whereas owner-occupiers consider their homes an investment for the future. Owner-occupiers often distinguish their house from that of another by making small changes, a style of doorframe or door way or perhaps different window frames. They often choose to paint their woodwork white, a conspicuous non-colour in a neighbourhood where doors of the rental houses are painted red, orange or yellow.

Owner-occupiers are proud of their homes and maintain them, something the elderly tenants also do, health permitting. A row of maisonettes, whose backyards face parking lot P1, have well-maintained gardens to the rear and poorly maintained entrances facing the Migrant Block.

Owner-occupiers of the Nice and Quiet Block are mainly native-born and aged 60 years plus. The women there went to a lower secondary school, while the men, who are generally more educated, are held responsible for the financial maintenance of the household. These residents have lived a long time in this cluster and feel responsible for the appearance of their homes, maintaining them well.

Tenants are mainly native-born and aged 55 plus or young migrant families who have recently settled. The tenants are often from a low-educated background and have poorly paid employment such as factory work. These tenants appreciate the presence of the large number of elderly native-born residents and the quiet living environment. Moreover, many maintain their houses to a high standard, especially the rear gardens. It may be tentatively suggested that tenants’ behaviour is to a large extent positively influenced by the behaviour of the owner-occupiers. In this cluster, tenants as well as owner-occupiers mingle. They often participate in conversation, provide help if needed and contribute to social control. In this cluster, residents have mainly strong ties, but weak ties also exist. Contact between owner-occupiers is superficial: they meet mainly in public spaces and avoid discussing personal issues. The social ties are mainly of a sociable nature and go together with social control practices.

To sum up, the owner-occupiers in the Nice and Quiet Block are harmonizers who have lived for a long time in the street and employ values and norms concerning the appearance of the street, its gardens and houses. For this purpose, they link themselves with neighbours and other residents in order to regulate behaviours and habits. This enables everyone to comply with their ideas of neatness and cleanliness. Here, there is no disaffiliation of the middle class; instead, successful attempts are employed to civilise the tenants. Home-making practices are combined with social contacts, also with non-locals. Here, notions of place belongingness overlap, partly attributed to the cluster and partly combined with the world outside the neighbourhood. The older residents—who distinguish themselves also by the physical appearance of their house—have set the norms and values applying to home-making practices in public spaces and others adjust to them, creating a sense of belonging; however, this does not apply to the sections of the cluster facing the Migrant Block, which are considered ‘unclean’ in reference to the poorly maintained public space. The politics of belonging demarcate the cluster in such a way that only those who adjust to the norms and values of the established residents will be included; others will be seen as a buffer towards the nearby Migrant Block.

### Tenants Island

Tenants Island is located at the bottom of Fig. 1 where it is physically segregated from other parts of the street. Here, four apartment blocks with an identical exterior are located: grey with whitewashed doors and yellow window frames. The ground and first floors consist of small housing units, while maisonettes can be found on the second and third floors. Originally, these housing units were meant for elderly people; however, at present a large number of migrant households live on the upper floors, while on the ground level and on the first floor, there are mainly ‘white’ single elderly, young adults and middle aged. All have a low income but differ in age and stage of life.

Young people often use these houses as a stepping-stone for better housing, but the elderly have no desire to leave their homes having lived there for a long time and formed an attachment to both their home and neighbourhood. Although the length of their stay may be limited, many interviewed tenants consider it important to maintain their garden properly.

Residents of Tenants Island still keep one eye on the streets, but social control is less prevalent now than it was in the past, possibly due to the increased levels of diversity of the resident population. The elderly still keep an eye on each other, but the younger generation makes less effort than the elderly to control neighbours’ unruly behaviour. There are a declining number of people willing to comment on irritations such as noise disturbance caused by the thin walls between houses. Often such annoyances are suppressed for a long period and result in an eruption and frictions coming to the surface ending in neighbours quarrelling.

The tenants maintain their houses and gardens well, and consider norms of neatness to be of great importance and believe themselves to be very good tenants. Here, weak ties among residents are dominant.

In sum, tenants reside only with other tenants, and operate independently of residents in the other clusters. Here, it is the physical demarcation of Tenants Island that determines certain isolation with tenants only contacting other tenants in the same block. With the exception of younger people, place attachment is high. However, contacts between most residents are based on weak ties. The elderly attempt to maintain public space in a neat and orderly fashion, but mutual intolerance concerning neighbours is manifest. These frictions between tenants lead to declining levels of shared home-making practices and a sense of place belongingness. Moreover, in this micro-setting, the politics of belonging are poorly developed. Demarcations between us and them in another cluster are rarely made due to a lack of common ground in this cluster.

### Owner-occupiers Island

This cluster located between the White Block and the Migrant Block has owner-occupiers who differ from those in the white houses on the other side of the street. Their houses are older and have brick walls. Most owner-occupiers are middle-aged married couples of native-Dutch origin; some have young children.

These homeowners have deliberately chosen a house in a quiet, peaceful neighbourhood with a green environment to the front of their houses and a backyard for their children to play in. In the streets traffic is almost entirely absent, and rubbish is put neatly into rubbish bins. Residents said that they had settled here because the neighbourhood appeared quiet. One migrant resident reported that she had ended up sending her children to a day care centre near to her work in another city. Originally, she had sent her children to day care in the neighbourhood, but became dissatisfied with the standard of cleanliness and the fact that all the children present were ‘foreigners’.

When neighbours meet—which rarely they do—they talk about superficial things such as the weather, children or happenings in the neighbourhood. Residents of Owner-occupiers Island report exhibiting different behaviour compared to their neighbouring clusters. They tend having mutually weak ties, are not very attached to the neighbourhood and complain about the violence that takes place nearby. These residents tend to live separate lives resulting in a lack of a sense of community, which is also due to the block in which they reside. Owner-occupiers are mainly middle aged with young children, work-oriented and lack attachment to the neighbourhood.

These owner-occupiers are not overly bothered by the residents of other clusters. Most are more externally oriented and have very limited contact with fellow neighbourhood residents, if indeed any. A resident suggests that distinguishing tenants and owner-occupiers should be done carefully:

In some flats there are tenants who are a bit antisocial, but (…) some are not. You cannot generalise about an entire group of people. Yes, that also applies to the owner-occupiers. There are also some antisocial owner-occupiers, maybe fewer of them, but still. When houses are purchased, the surrounding neighbourhood remains quiet. With owner-occupied houses it is always quieter. When people have children they will almost certainly buy a house. This happens very often.

To sum up, residents in this cluster appear to live in a place with little contact between residents either in or outside their cluster, if indeed any at all. There is a sharp distinction between where these externally oriented residents live and where they conduct their social contacts and work. This housing block is considered a place to live but rarely a place to socialise. Residents use their house for practical purposes, a place to rest after work, somewhere to sleep and are disinterested in contact with fellow residents. Here spatial belonging is privately organised. The high-rise building offers a spatial separation from the other clusters; as such, the space tends to lack a social demarcation. Here the politics of belonging are only supported by construction and not by social interaction.

### Migrant Block

The name of this cluster reflects the domination of its migrant population. Here, mainly less well-educated tenants of migrant origin and highly educated ‘white’ owner-occupiers are settled. Furthermore, some owner-occupiers of migrant origin have bought their rental unit here. Ineke (53), one of the ‘white’ highly educated owner-occupiers, who has been living here for 20 years, reports on the changes that have taken place:

Well, our neighbour had lived here for forty years and my sister used to live on the corner of this block, but she left. She couldn’t stand this neighbourhood any longer. (…) We had good contacts with them. Then a family from Somalia with eleven children settled here and the elderly man next door had a brain haemorrhage. So he and his wife moved out. They had to. And again, a foreign family moved in. Because of that, all the old people wanted to move and we got only foreign families in return. It happened very quickly. If all the old people had stayed, it would have been okay, but they wanted to leave and now we’ve got a family with eleven children who don’t care about anything. (…) I think they’re just antisocial. I’ve had many conflicts with them. Now I’m tired of struggling, so I have given up. Moreover, my neighbour has also given up. Now, I just call the police when something bothers me.

In this cluster, rental housing dominates and narratives tell us that mingling between tenants and owner-occupiers is rare. Here, separate rows of owner-occupied and rental housing exist with only three rows consisting of mixed owner-occupied and rental housing units.

The tenants can be divided into two groups: firstly, migrants, especially women who lead a secluded life and seem to be disinterested in the neighbourhood; and secondly, young enthusiastic tenants who want to invest in improving the liveability of their street and neighbourhood.

As shown above, two groups of owner-occupiers can be distinguished in this cluster. They can be categorised as: (1) disillusioned owner-occupiers—mainly native-born—who bought a house in the neighbourhood and have lived there for quite some time; and (2) former tenants—mainly Turkish-Dutch and Moroccan-Dutch—who have become owner-occupiers through having first rented a housing unit which they later purchased. The disillusioned owner-occupiers report that they used to participate in residents’ meetings together with tenants. These meetings were previously held in the neighbourhood centre for the purpose of talking through problems in the neighbourhood and determining possible solutions. However, today these owner-occupiers refrain from participation and feel that they are being neglected and ignored. On the basis of daily experiences, the ‘white’ owner-occupiers in this cluster have now formed a negative opinion of the neighbourhood and its tenants. The former tenants—mainly ‘whites’—are less negative. They have little contact with the ‘white’ owner-occupiers, but maintain contact with the tenants in this cluster.

The large gap between tenants and owner-occupiers is reflected in the behaviour and attitude of both groups. Tenants’ gardens are often not maintained and look messy; they have very high weeds and grass and the paths have weeds between the tiles. Meanwhile, the owner-occupiers usually keep their gardens clean and neat and take good care of their houses. These residents are proud of their property and show this by attention to detail; for instance, they might change a front door or window frames. Rental houses and gardens are badly maintained which may reflect the fact that the tenants, who are in the majority in this cluster, feel less responsible for the maintenance of their accommodation. Here, the role-model example set by owner-occupiers’ behaviour concerning houses and gardens has little or no impact on tenants’ behaviour. Interviewed owner-occupiers report that they want to move, not because of their houses but because of the neighbourhood. These residents feel they are stuck in the neighbourhood and are afraid that they will be unable to sell their house for a reasonable price, if at all.

In sum, the disillusioned ‘white’ owner-occupiers in this ethnically diverse cluster are proud of their residence but less happy with the living environment. They have experienced a hostile environment eventuating in mutual encounters and shared experiences among the owner-occupiers, whose ties, as a result, have since developed into sociable ones. Compared to the disillusioned owner-occupiers the former tenants tend to invest less in their physical living environment. These house buyers could be seen as the socio-economic ethnic climbers who have remained in the Migrant Block. Here, we see that these owner-occupiers tend not to disaffiliate themselves from others in their block because they are afraid of social closure.

Tenants who consider themselves inferior to the ‘white’ owner-occupiers include less well-educated migrants with children who can be roughly classified as women migrants, who live an isolated existence compared to the rest of the neighbourhood, while there is another group of young migrants involved in improving the living conditions and liveability of the neighbourhood. It looks as if the migrant population feels more at home here than the ‘whites’ who have been living here longer. Essentially, the ‘white’ owner-occupiers appear to have lost their sense of place belonging due to the changes in the living environment; they have had to cope with the arrival of large migrant families bringing with them different values, norms and lifestyles. Place attachment, including social involvement, has changed into a withdrawal from social life in the neighbourhood and thus a state of alienation. The diversity of residents in this cluster goes together with a weak politics of belongingness. Only owner-occupiers demarcate their boundaries by painting their doors differently from that of the tenants.

## Conclusion

If we look more closely at the everyday interaction between different types of tenants and owner-occupiers, two patterns occur. One pattern reflects the inward orientation of the yupps effectively strengthening the lower status premise among perceived inferior tenants. Here the combination of yupps and the perceived inferior tenants living together in the same street widens the gap between the groups. A second pattern reflects how tenants under the influence of harmonizers become closer to one another. Here bridges are constructed and shared norms and values are practised due to the authority of the owner-occupiers.

Social mixing can have a positive or negative impact on the liveability of the neighbourhood. A relatively equal status in a mixed cluster may help in establishing mutual contacts. Only where tenants have adjusted to the norms and values of the owner-occupiers can mutual contact lead to positive mutual judgement. This will not come about with an unequal status between owner-occupiers and tenants such as is found in the Migrant Block, where a widening gap exists between both groups, for example when white owner-occupiers place their children in schools outside the neighbourhood. Harmonizers have a positive influence on the malleable tenants, while the appearance of yupps and inferior tenants has a negative socialisation effect.

The spin off of a tenure mix needs a more differentiated approach in which the typologies of both groups should be distinguished. A crucial element in this regard can be found in the construction. The physical demarcation of blocks, wide streets and entrance locations of houses and gardens, influences contact between differing income groups. Once tenants or owner-occupiers are settled in a block, interaction with other blocks is far more difficult than if all houses were located in the same block or directly facing the houses of other blocks. Moreover, a high rise such as Owner-occupiers Island hinders contact among residents and the surrounding area.

It may be suggested that the micro-setting—including the surrounding environment, the physical position of the residence and how this influences middle-class disaffiliation—has a larger impact on lifestyle rather than on the nature of the entire neighbourhood. Moreover, homogeneity—in terms of ethnicity and/or social class—is the main predictor of neighbourhood relations in all clusters, which impacts upon home-making practices socially. Exceptions refer to where the owner-occupiers determine the norms and values in a mixed cluster or where tenants do not get on well together as in the Tenant Block. This case study has also shown that space–time configurations, in combination with specific kinds of residents, impact considerably upon home-making practices and senses of belonging, along with openness to diversity, all of which are not necessarily linked. This implies that mixing policies will not automatically lead to liveable neighbourhoods where everyone feels at home but can also add to segregation and exclusion along class and ethnic lines. Time also plays an important role in our findings. The longer the period the established dominate home making in a micro-setting the more residents feel at home. However, as the role of the established residents declines and new groups—e.g. migrants in the Migrant Block—arrive, the ‘former’ established become outsiders. Here diversity in the micro-setting hinders the creation of a shared sense of belongingness.

It is important to look at those who have the power to determine dominant kinds of contact, but also at *how* housing policies impact upon these. Only where there was a majority group of long-stay residents was it possible for home making and creating a sense of belongingness for everybody living in the micro-setting. Permanency of residents offers the possibility of stability concerning home-making practices. Once the majority consists of newcomers—yuppies and migrants—who make no attempt to communicate with the minority, a shared sense of belonging becomes absent, which can lead to tectonic relationships within and between blocks. Here processes of discontinuity of home-making practices go hand in hand with hostility and alienation, which in turn can harm senses of belonging and aims of a tenure mix. This study has shown that a combination of place belongingness and practices of belonging provide a more in-depth analysis rather than focussing only on the ‘neutrality’ of home making and senses of belonging. In other words, local micro-politics of belonging provide insight into the mechanisms of inclusion and exclusion.

## Footnotes

1 During the period of research, these migrants were called allochthones in the Netherlands if at least one of the parents is born abroad. In this paper the concept is expressed by the word ‘migrant’.
